# The development and use of a pharmacist-specific Mini-CEX for postgraduate year trainees in Taiwan

**DOI:** 10.1186/s12909-019-1602-2

**Published:** 2019-05-22

**Authors:** Chung-Jen Wei, Tzu-Hsuan Lu, Shu-Chen Chien, Wan-Tsui Huang, Yueh-Ping Liu, Cho-Yu Chan, Chiung-Hsuan Chiu

**Affiliations:** 1 Department of Public Health, Fu Jen Catholic University, New Taipei City, Taiwan; 2 Medical Quality Department, Taipei Medical University-Shuang Ho Hospital, New Taipei City, Taiwan; 3 School of Pharmacy, College of Pharmacy, Taipei Medical University, Taipei, Taiwan; 4 Department of Pharmacy, Taipei Medical University Hospital, Taipei, Taiwan; 5 Department of Pharmacy. Cathay General Hospital, Taipei, Taiwan. School of Pharmacy. Taipei Medical University, Taipei, Taiwan; 6 Department of Emergency Medicine, National Taiwan University Hospital, Taipei, Taiwan; 7 Changhua Christian Hospital, Chunghua, Taiwan

**Keywords:** On-the-job training, Work-based assessment, Postgraduate year training program, Mini-CEX, PGY trainees

## Abstract

**Background:**

Clinical pharmacists must have a complex combination of academic knowledge and practical experience that integrates all aspects of practice. Taiwan’s Ministry of Health and Welfare in 2007 launched the Postgraduate Year (PGY) training program to increase the standard of pharmaceutical care. This study aims to develop a pharmacist-specific Chinese-language Mini-Clinical Evaluation Exercise (Mini-CEX) to evaluate the professional development of postgraduate year trainees.

**Method:**

The specialized Mini-CEX was developed based on the core competencies of pharmacists, published literature, and expert opinion. A pilot test and evaluator workshop were held prior to the administration of the main test. Fifty-three samples were recruited. The main study was conducted at two regional teaching hospitals and a medical center teaching hospital in Taiwan between February and June 2012. The results were analyzed with the kappa statistic (inter-rater reliability) and descriptive statistics, while the Kruskal-Wallis test was used to examine the PGY trainees’ Mini-CEX scores based on their performances.

**Results:**

Trainees who had recently completed PGY programs (C-PGY) and 2nd year PGY trainees (PGY2) earned excellent scores, while the 1st year PGY trainees (PGY1) earned satisfactory scores in overall performance. C-PGY and PGY2 trainees also performed significantly better than PGY1 trainees in the organization and efficiency domain, and the communication skills domain.

**Conclusion:**

This study demonstrates the feasibility of using the newly developed pharmacist-specific Chinese-language version of the Mini-CEX instrument to evaluate the core competencies of PGY trainees in clinical settings.

**Electronic supplementary material:**

The online version of this article (10.1186/s12909-019-1602-2) contains supplementary material, which is available to authorized users.

## Background

Over the past few decades, the roles and obligations of hospital pharmacists around the globe have changed drastically. The healthcare industry places an ever-increasing emphasis on clinical work as a method for improving the safe, effective, and synergistic use of medication [1]. Clinical pharmacists have many responsibilities, including healthcare promotion, treatment of disease, and ensuring rational usage of pharmaceuticals by both healthcare professionals and patients.

According to the American College of Clinical Pharmacy, the competencies of clinical pharmacists are: to integrate therapeutic knowledge, problem-solving skills, judgment, and attitudes into service to meet the rising expectations of their teams and patients; to educate and communicate effectively with patients and healthcare professionals to ensure optimal patient outcomes; to maintain a continuously expanding and up-to-date knowledge base; to contribute to the development and implementation of protocols and critical pathways to manage patient populations; and to possess a therapeutic knowledge base of sufficient breadth and depth to effectively promote rational medication use [2]. These are the core competencies of appropriately educated pharmacists working within healthcare facilities. However, recent graduates often struggle to successfully translate theory into practice [3]. It is a crucial goal of new employee orientation and training to ensure that all entry-to-practice pharmacists can provide high-quality service. Bridging the gap between the required and equipped competencies of today’s pharmacy graduates upon entry into the workplace is a prominent concern.

To ensure pharmacists’ competence in all aspects of practice in order to provide holistic and patient-centered care, the Taiwanese government in 2007 launched the postgraduate year (PGY) program [4]. The Ministry of Health and Welfare also provided partial reimbursement to teaching hospitals to conduct training for 16 different groups of healthcare professionals, including pharmacists, nurses, and physical therapists, through the “Instruction Fee Reimbursement Program for Teaching Hospitals” to regulate on-the-job training. These programs are managed by the Joint Commission of Taiwan (JCT) under the supervision and instruction of the relevant professional associations. The purpose of the JCT PGY pharmacy program is to enhance the quality of comprehensive medical care by ensuring the acquisition of therapeutic knowledge, skills, attitudes, and behaviors over the course of the supervised 24-month training program. The program aims to equip trainee pharmacists with (1) proficient medication knowledge and evidence-based skills; (2) a patient-centered and total care attitude; (3) professional ethical reasoning and communication skills; and (4) ability to work in a team [5]. The teaching hospitals involved in the program develop their own training systems based on these primary objectives.

The American Board of Internal Medicine developed a focused, brief, and observed clinical encounter called the Mini-Clinical Evaluation Exercise (Mini-CEX). This encounter allows evaluators to directly observe the performance of a clinical task, and immediately follow up with a consultation between the trainee and evaluator, during which the evaluator completes an assessment and provides the trainee with feedback on their performance [6]. With the benefits of immediate constructive feedback and ongoing evaluation, the Mini-CEX is recognized as a useful tool for professional development and evaluating training program results, leading to improved clinical performance [7]. The Mini-CEX has been recognized by the Joint Programmes Board as a feasible and reliable method to assess the effectiveness of postgraduate pharmacy education [8].

Due to the effectiveness, reliability, and convenience of the Mini-CEX, it has been very popular for the evaluation of medical interns, residents, and other medical professionals in many Western countries as well as in Taiwan [9, 10]. The role of pharmacists in the inpatient setting has expanded to emphasize client-centered and multi-disciplinary care. Although physicians and pharmacists often collaborate and share some common ground on patient care, many of the required competencies, attitudes, and practices differ significantly. Pharmacists are expected to play an active role in interprofessional care, while drawing on the knowledge, skills, and practices specific to their discipline [11], therefore, the direct application of the physician Mini-CEX to pharmacists is inappropriate.

There are a few pharmacist-specific Mini-CEXes, such as those developed by the Royal Pharmaceutical Society of Great Britain [12], the Department of Pharmacy at National Singapore University [13], and the Competency Development and Evaluation Group [14]. These tools focus on patient care, problem solving, and personal practice, but new pharmacists at hospitals in Taiwan are expected to perform more basic tasks, such as dispensing medication and providing consultation/education, while pharmacy divisions in most major hospitals deal with an average of 7000 outpatient visits per day. Only senior trainees are allowed to provide clinical pharmacy services after at least six months of PGY training. A work-based assessment of new pharmacists’ core competencies that suits Taiwan’s context is needed. Therefore, this study aimed to develop a Chinese-language Mini-CEX instrument that evaluates the professional development of pharmacists in PGY training programs in Taiwan.

## Methods

### Study design

This study was conducted to assess the competencies of new pharmacists participating in PGY training programs. A Chinese-language Mini-CEX was devised, tested, and utilized in six phases: (1) After a literature review and expert interviews, the instrument was developed; (2) An initial workshop for evaluators was held prior to the pilot test to insure inter-rater reliability; (3) The content of the instrument was re-evaluated and modified after a pilot test was conducted using a group of PGY trainees; (4) Workshops were held for evaluators before the main PGY trainee assessment; (5) A cross-sectional survey was conducted to measure the competencies of PGY trainees from the perspective of evaluators; (6) The core competencies of trainee groups were assessed and compared.

### Instrument development

The specialized Mini-CEX for pharmacists was developed based on the core competencies of pharmacists outlined by the Canadian National Association of Pharmacy Regulatory Authorities [15]*,* the accreditation standards for pharmacy residencies set by the American Society of Health-System Pharmacists (ASHP) [16], the clinical pharmacist competencies released by the American College of Clinical Pharmacy [2], and the pharmacist training guide produced by the Japan Pharmacists Education Center [17]. The ladder system established by the Taiwan Society of Health-System Pharmacists (TSHSP) [18] was also incorporated into the evaluation criteria. Nine core competencies were initially included in the instrument, which was validated by five experts: two MD/PhDs specializing in instrument development and three PharmD-qualified directors of hospital pharmacy departments. Two rounds of the Delphi method were applied to check the face validity and the allocation of sample behavior items to the appropriate domains [19]*.* A three-point rating system was utilized to decide whether to include domains: domain is important and should be retained (3 points), domain is important but needs revision (2 points), and domain is not important and must be removed (1 point). The domains that scored an average of at least 2 points were retained.

The final version of the Mini-CEX included nine domains: pharmacology knowledge, patient care knowledge, consultation skills, professional health education skills, management of drug distribution, organization and efficiency, professionalism, communication skills, and overall performance (shown in Additional file 1). All descriptive items for each domain were adapted from guidelines provided by the ASHP, translated into Chinese, and validated using the Delphi method.

The complete evaluation form consisted of the evaluators’ and trainee’s identities, number of years of PGY training, the date and place of the evaluation, detailed descriptions of each domain, observations and feedback time, and the overall score. Descriptions of each domain are included in Additional file 2.

Trainees were scored based on the qualities of a “competent” pharmacist (equivalent to stage 3 of the TSHSP ladder system) [18, 20]. Each core competency was rated on a 1–9 point scale as unsatisfactory (1–3), satisfactory (4–6), or excellent (7–9). Unsatisfactory scores (1–3) were defined as “extremely poor” (1 point), “poor” (2 points), and “nearly passing” (3 points). Satisfactory scores (4–6) were defined as “meets minimum expectations” (4 points), “average” (5 points), and “slightly above average” (6 points). Excellent scores (7–9) were defined as “meets most expectations and exceeds all others” (7 points), “exceeds most expectations and meets all others” (8 points), and “exceeds all expectations” (9 points). All behaviors were rated based on the observed practice for the selected case. Evaluators were advised to choose “not applicable” when certain behaviors were not performed.

### Participants and setting

Since this study focused on the assessment of trainees based on the input of evaluators, two independent sets of evaluators and trainees were recruited for the pilot test and the main study. The qualifications required of evaluators and trainees were based on the requirements of the PGY program in Taiwan. Evaluators were required to have senior pharmacist status, at least four years of working experience in a teaching hospital, and instructor certification. The inclusion criteria for PGY trainees were: current participation in a PGY program or completion of PGY training within a year of the start of the study, and current employment in the pharmacy department of a teaching hospital.

### Pilot study and evaluator workshop

The pilot test was held at two regional teaching hospitals and a medical center teaching hospital in Taiwan in December 2011 to confirm the appropriateness of the instrument. Thirteen PGY trainees and eight evaluators were recruited. Ten trainees were female (76.9%) and three were male (23.1%). The trainees reported a mean age of 24.38 (SD 2.21) and mean tenure of 6.33 months (SD 4.98) in PGY programs.

A two-session workshop was held for the evaluators prior to the pilot study to finalize the instrument and maintain the consistency of ratings for the same items between multiple respondents [21]. In the first session, the background, concept, purpose, and procedure of the Mini-CEX were reiterated. In the second session, a sample video clip of a pharmacist-patient Mini-CEX encounter was shown to the evaluators, who were asked to give a score for the encounter and provide a brief explanation to the other evaluators for the score given. An evaluator-hosted discussion was then held to establish performance standards and reach a consensus among the evaluators. Once a consensus was reached, the video clip was shown to the evaluators again for scoring. After analysis of the second-round evaluation results, the inter-rater reliability was determined.

## Main study

The main study was conducted at two regional teaching hospitals and a medical center teaching hospital in Taiwan between February and June 2012. Fifty-three PGY trainees and 22 senior pharmacist evaluators were recruited using the same inclusion criteria as the pilot study, with an evaluator to trainee ratio of 1:2.4.

All evaluators followed the protocol outlined in the workshop. A trainee’s performance was observed by two evaluators who rated them as compared with a TSHSP stage 3 pharmacist. One evaluator was a direct supervisor, while the other was a mentor of the trainee. These relationships were chosen to minimize subjectivity and enhance the quality of the assessment [22]. All behaviors were rated based on the observed practice in the selected settings, including dispensing medication, providing clinical pharmacy services, and giving a medication consultation. Technical skills, knowledge, and interaction with patients and peers were assessed. After asking the trainee for therapeutic decisions and reminders for therapeutic management, the evaluators completed the Mini-CEX form and provided “sandwich” feedback followed by direct instructions for improvement [23].

### Data analysis

The results were analyzed in two parts using SPSS for Windows 22.0. Firstly, the kappa statistic (inter-rater reliability) was calculated based on the scores given by the evaluators before and after the discussion during the workshop. Secondly, descriptive statistics and the Kruskal-Wallis test were used to examine the PGY trainees’ Mini-CEX scores.

### Ethics approval

The Joint Institutional Review Boards of participating hospitals approved the study. The faculty members and PGY trainees were informed of the purpose of the study and assured of confidentiality. Written informed consent was obtained from the enrolled participants for purposes of publication of this report. The Joint Institutional Review Board of Taipei Medical University approved the study. The approval number is TMU 201012008.

## Results

### Validity and reliability

In this study, face validity was assessed by five experts. A two-round Delphi process was conducted to devise the instrument. After the evaluators’ workshop, the inter-rater reliability of the instrument was obtained on the basis of the second-round evaluation. The inter-rater reliability of the instrument was 0.7.

### Descriptive statistics

The main study proceeded with 53 PGY trainees, including 1st year (PGY1) and 2nd year (PGY2) trainees, and pharmacists who had completed their PGY training within the past year (C-PGY). A total of 104 evaluations were collected with a 100% response rate (Table 1). The trainees had completed an average of 14.7 months of training, and PGY1 students comprised the largest proportion of the sample (47.2%). They were mostly female (79.2%), and the most common age group was 25 to 29 years (64.2%). Trainees were observed dispensing medication for outpatients (55.4%), dispensing medication for hospitalized patients (18.5%), and providing clinical pharmacy services (4.6%).

**Table 1:** Descriptive Statistics of PGY Trainees

	Variable	n (%)
Duration of Training 14.7 (11.3)	1st year (PGY1)	25 (47.2)
	2nd year (PGY2)	20 (37.7)
	Recent graduate (C-PGY)^a^	8 (15.1)
Gender	Male	11 (20.8)
	Female	42 (79.2)
Hospital^b^	A	9 (17.0)
	B	8 (15.1)
	C	36 (67.9)
Age 25.5 (1.9)	21–24	15 (28.3)
	25–29	34 (64.2)
	Above 30	2 (3.8)
	Not reported	2 (3.8)

Note: Mean (SD); ^a^completed PGY training within the past year

^b^A (medical center); B (regional hospital); C (medical center)

The mean ratings for all 53 trainees were highest in the communication skills domain (7.26 ± 1.20) and lowest in the organization and efficiency domain (6.79 ± 1.16). The mean score in the overall performance domain was 7.07 (±1.17), indicating that the competencies of trainees enrolled in the study met the criteria for “excellent” performance (Table 2). In the subgroup analysis, PGY1 trainees performed best in pharmacology knowledge (6.94 ± 1.03), PGY2 trainees performed best in communication skills (7.82 ± 0.86), and C-PGY trainees performed best in medication consulting skills (7.75 ± 1.04).

**Table 2:** Cross-Comparison of PGY Groups on Evaluation Dimensions

Evaluation Dimensions	Overall (*N* = 53)	PGY1 (*n* = 25)	PGY2 (*n* = 20)	C-PGY (*n* = 8)	*p* value	Post-hoc
Pharmacology Knowledge	7.1 (0.9)	6.9 (1.0)	7.5 (0.4)	7.1 (0.3)	.12	
Patient Care Knowledge	7.1 (1.0)	6.6 (1.0)	7.4 (0.9)	7.3 (0.9)	.08	
Medication Consulting Skills	7.0 (1.0)	6.6 (1.1)	7.0 (1.4)	7.7 (1.0)	.17	
Health Professional Education Skills	6.8 (1.6)	6.5 (1.1)	7.0 (1.5)	7.2 (1.0)	.42	
Management of Drug Distribution	6.9 (1.1)	6.5 (1.2)	7.1 (1.4)	7.3 (0.6)	.13	
Organization and Efficiency	6.7 (1.1)	6.2 (1.1)	7.1 (1.2)	7.2 (0.7)	.03	PGY2 > PGY1*; C-PGY > PGY1*
Professionalism	7.0 (1.1)	6.5 (1.2)	7.5 (0.8)	7.5 (0.8)	.07	
Communication Skills	7.2 (1.0)	6.6 (1.2)	7.8 (0.8)	7.5 (1.1)	.00	PGY2 > PGY1*
Overall Performance	7.0 (1.1)	6.5 (1.3)	7.4 (0.8)	7.6 (0.6)	.01	PGY2 > PGY1*; C-PGY > PGY1*
Observation Time	14.3 (10.1)	12.9 (7.6)	17.0 (13.7)	12.5 (5.1)	.40	
Feedback Time	6.7 (4.1)	7.5 (4.6)	6.3 (4.4)	5.6 (0.8)	.58	

Note: Mean (SD), C-PGY = Completed PGY training within the past year

A one-way ANOVA shows that C-PGY trainees performed significantly better (*P <* 0.05) than PGY1 trainees in the organization and efficiency domain, and the overall performance domain. Similarly, PGY2 trainees performed significantly better (*P <* 0.05) than PGY1 trainees in organization and efficiency, communication skills, and overall performance.

## Discussion

The PGY training program is beneficial to the development of optimal pharmaceutical care for clinical pharmacists in Taiwan, as it encourages the integration of academic knowledge and clinical practice. This is especially important for new pharmacists who are entering the workforce, facing real patients, and taking responsibility for their clinical skills and decisions for the first time [24]. As healthcare development advances, the importance of continuous education and updating the undergraduate and graduate knowledge bases increases.

The study instrument included nine major competencies described by major professional pharmacy associations, and a two-round Delphi process was utilized to verify the face validity. The Delphi method is a feasible way to reach consensus, especially for pharmacist competency studies [25]. The nine competency domains listed in the Mini-CEX represent the skills pharmacists must have. They also align with the ladder systems for clinical pharmacists and the Good Pharmacy Practice guidelines [18, 26]. These guidelines, released by the TSHSP, describe the standards used to assess the quality of pharmacy practice and provide the basis of current and future practice-support initiatives.

The Mini-CEX is a recognized evaluation method with strong validity [27] that has frequently been utilized for both interns and residents across medical specialties. As opposed to the written evaluations that typically bookend the PGY program, this assessment of actual patient encounters through direct observation can provide ongoing data on trainee performance. The Mini-CEX is a workplace-based assessment that fosters the type of self-directed learning environment essential for continuing professional development [28]. A Mini-CEX typically includes 15 min of observation and 5 min of feedback [6]. In our study, the mean observation and feedback times were 14.30 and 6.79 min, respectively. The feedback time was relatively long due to its structured nature, as evaluators were instructed to provide detailed recommendations, and trainees were encouraged to respond. There was no significant difference in feedback times between the three groups. The provision of feedback after direct observation is a critical component of deliberate practice, a learning strategy believed to maximize the improvement of clinical competency through repetition, guidance, and coaching [29].

There were eight evaluators in the evaluator workshop, and the instrument showed acceptable reliability (inter-rater reliability of 0.7). Although the physician Mini-CEX has been proven to successfully differentiate between competency levels with appropriate reliability and construct validity [30], some studies have demonstrated limitations of the Mini-CEX due to significant inter-evaluator variability [31]. Medical educators typically evaluate trainees based on personal experience and performance. Formal training is needed to minimize the variability between evaluators and to reach consensus. A study by Liao et al. demonstrated that faculty participation in Mini-CEX workshops strengthened the consistency of evaluators and resulted in the implementation of a successful Mini-CEX assessment program [32]. Meanwhile, a study by Arora et al. found that using standardized video-based scenarios depicting differing levels of performance, communication, and professionalism in a variety of settings ensured valid and reliable physician handoff Mini-CEX [33]. Moreover, a meta-analysis of non-medical education studies suggested that training workshops for raters were associated with score accuracy improvement. This is also important for evaluator professional development, as the literature has highlighted positive impacts on personal learning and performance from serving as a Mini-CEX evaluator [34].

The goal of assessing the core competencies of PGY trainees, as demonstrated by how they interacted with patients during clinical encounters, was achieved using a customized pharmacist Mini-CEX. The results for each trainee can be considered with regard to the learning curve and desired teaching outcomes of their program. Based on the individual results, trainees and teachers can adjust their approaches to lessons, behavior, and attitudes to achieve better learning outcomes.

The results of this study revealed the successes of PGY training programs at all three participating institutions. The average scores in the “overall performance” domain for all trainee groups were high: average PGY1 scores were “satisfactory,” while average PGY2 and C-PGY scores were “excellent.” Notably, every C-PGY trainee scored a 7.0 (the threshold for an “excellent” score) or higher in overall performance. This was consistent with the results of other studies evaluating PGY trainee training outcomes in Taiwan [35].

Mean scores for all nine competency domains were higher for C-PGY trainees than PGY1 pharmacists. In addition to the overall performance domain, two other domains showed statistically significant higher scores for the senior groups: the organization and efficiency domain, and the communication skills domain. These results demonstrate the importance of postgraduate on-the-job training and indicate that the development and enhancement of these abilities requires actual practice in the workplace, such as interaction with other medical professionals and participation in multidisciplinary teams.

Our study reveals significant differences in organization and efficiency, as well as communication skills of trainees undertaking PGY programs. Based on the outcomes of the JCT’s 24-month PGY training program, organization and efficiency, as well as communication skills could be improved with training in multidisciplinary teamwork, as well as social and administrative pharmacy [5]. In most instances, the trainees could enroll in these elective courses only if they were already performing well after basic training and the courses are provided by qualified faculty. We suggest that social and administrative pharmacy, as well as interprofessional practice be listed as required subjects in the Taiwanese PGY training program, as it is in many pharmacist residency programs in the United States [10]. In the meantime, pharmaceutical education policy should encourage teaching hospitals to improve the quality of instruction in required courses to facilitate better patient care and faster acquisition of clinical skills.

### Limitations

This study has three limitations: First, the research was conducted at one regional hospital and two medical centers in northern Taiwan. Although these institutions were teaching hospitals accredited by the JCT, the quality of teaching and the format of the PGY program differs between hospitals and regions. The performance of pharmacists throughout the national PGY program requires further evaluation. Second, this cross-sectional study only represents the ability of trainees at the time of the study. To evaluate individual growth through participation in the PGY program, advanced longitudinal studies are required. Third, a major advantage of formative evaluation methods in training programs is their ability to help modify clinical practice following the evaluation and feedback process. Unfortunately, the changes in knowledge and behavior following the Mini-CEX were not able to be measured as part of this study.

## Conclusion

This study developed a Chinese-language PGY trainee version of the Mini-CEX instrument. The instrument was based on the structure of the previously validated and widely used Mini-CEX for medical residents [6]. The specific elements of this Mini-CEX for pharmacists were developed based on clinical practice and educational guidelines, published literature, and expert opinion. This process resulted in nine domains for PGY trainee assessment: pharmacology knowledge, patient care knowledge, medication consulting skills, professional health education skills, management of drug distribution, organization and efficiency, professionalism, communication skills, and overall performance. We believe that these adjustments have created an assessment tool that is more compatible with the core competencies of pharmacists. It should serve as a practical workplace-based assessment for educators to evaluate a trainee’s strengths and weaknesses, and to give timely formative feedback.

The results demonstrate that the Mini-CEX is a feasible tool to evaluate the professional development of pharmacists. Trainee scores indicated the merit of their institutions’ PGY training programs. Additionally, the senior trainees performed significantly better in the core competencies of overall performance, organization and efficiency, and communication skills, which demonstrates the importance of post-graduate onsite training and interaction with other health professionals. Multidisciplinary team participation, and social and administrative pharmacy should be listed as required subjects in the Taiwanese PGY training program.

Our Mini-CEX is feasible for use in clinical settings for the evaluation of the core competencies of PGY trainees. Nevertheless, further longitudinal studies using this tool are recommended to assess personal growth and the development of expertise during the PGY training period and beyond.

## Additional files

Additional file 1: Pharmacist Mini-CEX Evaluation Sheet. (DOCX 23 kb)

Additional file 2: Definitions of major domains. (DOCX 16 kb)

## Abbreviations

ASHP: The American Society of Health-System Pharmacists
C-PGY: Those who have completed their PGY in the past year
JCT: Joint Commission of Taiwan
Mini-CEX: Mini-Clinical Evaluation Exercise
PGY: Postgraduate Year
PGY1: First-year postgraduate trainees
PGY2: Second-year postgraduate trainees
TSHSP: The Taiwan Society of Health-System Pharmacists
