# Supplementary material for: The development and use of a pharmacist-specific Mini-CEX for postgraduate year trainees in Taiwan
Source: BMC Med Educ. 2019 May 22;19:165. doi: 10.1186/s12909-019-1602-2 (PMC6530012; doi:10.1186/s12909-019-1602-2)
Supplement: Supplementary file 1 — Pharmacist Mini-CEX Evaluation Sheet. (DOCX 23 kb) [file 12909_2019_1602_MOESM1_ESM.docx]

Pharmacist Mini-CEX Evaluation Sheet

| Evaluator： | | | | | | Date： / / / | | | | |  |
| --- | --- | --- | --- | --- | --- | --- | --- | --- | --- | --- | --- |
| Trainee： | | | | | | Position：□PGY1 □PGY2 □C-PGY | | | | |  |
| Setting：□Medication delivery for outpatients □Dispensing for outpatients  □Dispensing for hospitalized patients □Clinical Pharmacy Services □Other | | | | | | | | | | |  |
| Items | Not Applicable | Grading | | | | | | | | | |
|  |  | Unsatisfactory | | | Satisfactory | | | | Excellent | | |
|  |  | 1 | 2 | 3 | 4 | | 5 | 6 | 7 | 8 | 9 |
| Pharmacology Knowledge |  |  |  |  |  | |  |  |  |  |  |
| Patient Care Knowledge |  |  |  |  |  | |  |  |  |  |  |
| Medication Consulting Skill |  |  |  |  |  | |  |  |  |  |  |
| Health Professional Education Skill |  |  |  |  |  | |  |  |  |  |  |
| Management of Drug Distribution |  |  |  |  |  | |  |  |  |  |  |
| Organization and Efficiency |  |  |  |  |  | |  |  |  |  |  |
| Professionalism |  |  |  |  |  | |  |  |  |  |  |
| Communication Skill |  |  |  |  |  | |  |  |  |  |  |
| Overall Performance |  |  |  |  |  | |  |  |  |  |  |

Mini-CEX time：Observation: Minutes

Feedback: Minutes

Other Comments：

Evaluator： Trainee：

**Pharmacology knowledge**

- Pharmacy practice (e.g. pharmaceutical care, compounding & dispensing, structured practical training)
- Demonstrate additional skills that contribute to practicing effectively in the health care environment.
- Evaluate the appropriateness of the choice of drug

**Patient care knowledge**

- Develop a professional relationship with the patient
- Gather patient information and assess its relevance to patient care
- Identify a patient’s desired therapeutic outcomes
- Identify a patient’s actual and potential drug related problems
- Develop therapeutic plans, recommending therapeutic options, doses, scheduling/administration, required drug devices and, compliance aids
- Develop, implement, and fulfill plans to monitor the patient’s progress towards desired therapeutic outcomes
- Document information
- Provide evidence-based, patient-centered medication therapy management with interdisciplinary teams
- Cognitive service prescription evaluation

**Medication consulting skill**

- Identify sources of relevant information
- Provide information on disease prevention and health promotion
- Serve as an authoritative resource on the optimal use of medications
- Provide instruction for drug indication, food reaction, preservation techniques, and other precautions

**Health educating skill**

- Maintain an involvement in the education of pharmacy students, interns, and residents
- Demonstrate excellence in the provision of training or educational activities for health care professionals and health care professionals in training

**Management of drug distribution**

- Perform, supervise, and/or review drug preparation and distribution activities
- Manage situations involving drug diversion or inappropriate use

**Organization and Efficiency**

- Manage workflow within the dispensary and professional areas of the pharmacy
- Interpret and apply the drug utilization, reimbursement, and pharmacoeconomic policies of health care facilities and agencies as well as federal, provincial, and third party drug insurance plans
- Manage and improve the medication-use process

**Professionalism**

- Accept responsibility for actions and decisions
- Demonstrate respect for others
- Provide professional pharmacy care to individual patients that complies with the ethical guidelines governing the profession
- Appropriate inter-professional relationships required to provide quality pharmacy care to individual patients
- Undertake non-pharmacy practice-related activities that are consistent with, and do not take advantage of the influence of, their status as a health professional
- Reject offers of gifts or advantages that give the appearance of bias or conflict of interest

**Communication Skills**

- Demonstrate respect, sensitivity, and empathy when communicating with others
- Demonstrate appropriate verbal, non-verbal, and listening skills
- Effective patient-interviewing techniques including: initiating a session, exploring problems, understanding the patient’s perspective, structuring the discussion, building a patient relationship, facilitating the patient’s involvement, explaining, planning, and closing the discussion
