# Supplementary material for: The development and use of a pharmacist-specific Mini-CEX for postgraduate year trainees in Taiwan
Source: BMC Med Educ. 2019 May 22;19:165. doi: 10.1186/s12909-019-1602-2 (PMC6530012; doi:10.1186/s12909-019-1602-2)
Supplement: Supplementary file 2 — Definitions of major domains. (DOCX 16 kb) [file 12909_2019_1602_MOESM2_ESM.docx]

Appendix 2 Definitions of major domains

*Pharmacology Knowledge*: This domain primarily assesses a PGY trainee’s comprehensive knowledge of medications. It also addresses the trainee’s ability to provide the appropriate pharmaceutical care, perform compounding, and dispense medications in a form appropriate to the type of drug.

*Patient Care Knowledge*: This domain addresses a trainee’s ability to gather patient information, identify desired therapeutic outcomes, develop medication therapy management plans, and monitor progress after implementation of treatment plans. It also addresses cooperation with interdisciplinary teams.

*Medication Consulting Skills*: This domain appraises a trainee’s ability to discuss the effects and side effects of medications, as well as provide instructions and other precautions to ensure the optimal use of medications. The PGY　trainee should also provide information about disease prevention and individual health education during patient consultations.

*Professional Health Education Skills*: This domain addresses a trainee’s ability to participate in the instruction of pharmacy students, interns, residents, or other healthcare professionals to support optimal patient care.

*Management of Drug Distribution*: This domain evaluates a trainee’s ability to manage drug distribution appropriately by performing or supervising the acquisition, storage, preparation, and distribution of drugs to ensure the safety, accuracy, and quality of supplied products. It also evaluates their ability to address situations involving drug diversion or inappropriate use.

*Organization and Efficiency*: This domain tests a trainee’s ability to manage workflow within the pharmacy department, to utilize cost-effectiveness principles, and to improve the medication-use process.

*Professionalism*: This domain assesses a trainee’s ability to accept responsibility for their actions and decisions, to demonstrate respect for others, and to perform according to the law and the professional and ethical standards of pharmacists.

*Communication Skills*: This domain assesses the quality of a trainee’s patient interview and listening skills. Effective interviewing techniques include: initiating a session; exploring problems; understanding the patient’s perspective; structuring the discussion; building a relationship; facilitating the patient’s involvement; and explaining, planning, and closing the discussion.

*Overall Performance*: This domain evaluates the overall competency of the PGY trainees.
